# Anatomy of the Arteries of the Human Body, &c.

**Published:** 1862-10

**Authors:** 


ANATOMY OF THE ARTERIES OF THE HUMAN BODY, DESCRIPTIVE AND SURGICAL, WITH THE DESCRIPTIVE ANATOMY OF THE HEART. By JOHN HATCH POWER, M.D., Fellow, and Member of Council, of the Royal College of Surgeons; Professor of Descriptive and Practical Anatomy, in the Royal College of Surgeons; Surgeon to the City of Dublin Hospital, &c. Authorized and Adopted by the Surgeon-General of the United States Army, for use in Field and General Hospitals. Philadelphia: J. B. LIPPINCOTT & Co. 1862.

This is a small-sized octavo volume of 401 pages; printed on good paper, and excellent type. We have not had time to examine in detail the merits of this work. But it is, doubtless, accurate; and from its moderate size, and numerous cuts, it will be found a very convenient work for reference by the army surgeon, who is often deprived of all access to ordinary libraries.

For sale by S. C. GRIGGS & Co., 41 Lake Street, Chicago.

